# Mechanistic stratification in electroactive biofilms of *Geobacter sulfurreducens* mediated by pilus nanowires

**DOI:** 10.1038/ncomms12217

**Published:** 2016-08-02

**Authors:** Rebecca J. Steidl, Sanela Lampa-Pastirk, Gemma Reguera

**Affiliations:** 1 Department of Microbiology and Molecular Genetics, Michigan State University, 567 Wilson Road, Rm 6190, Biomedical and Physical Science building, East Lansing, Michigan 48824, USA

## Abstract

Electricity generation by *Geobacter sulfurreducens* biofilms grown on electrodes involves matrix-associated electron carriers, such as *c*-type cytochromes. Yet, the contribution of the biofilm's conductive pili remains uncertain, largely because pili-defective mutants also have cytochrome defects. Here we report that a pili-deficient mutant carrying an inactivating mutation in the pilus assembly motor PilB has no measurable defects in cytochrome expression, yet forms anode biofilms with reduced electroactivity and is unable to grow beyond a threshold distance (∼10 μm) from the underlying electrode. The defects are similar to those of a Tyr3 mutant, which produces poorly conductive pili. The results support a model in which the conductive pili permeate the biofilms to wire the cells to the conductive biofilm matrix and the underlying electrode, operating coordinately with cytochromes until the biofilm reaches a threshold thickness that limits the efficiency of the cytochrome pathway but not the functioning of the conductive pili network.

## Abstract (web summary)

The roles played by cytochromes and conductive filamentous appendages (pili) in the electrical conductivity of *Geobacter* bacterial biofilms are controversial. Here, Steidl *et al*. present evidence that both mechanisms cooperate in thin biofilms, while pili are important for conductivity across thicker biofilms.

## Graphical abstract

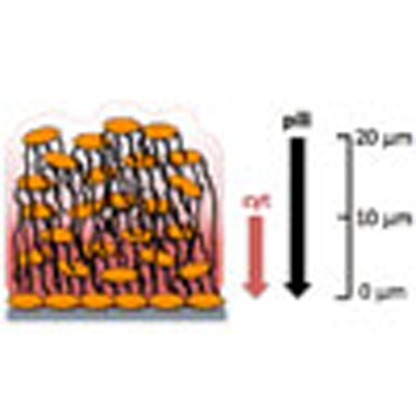

The ability of *Geobacter* bacteria to completely oxidize organic compounds to CO_2_ with an electrode poised at a metabolically oxidizing potential shows promise for the conversion of renewable biomass into electricity, hydrogen and/or liquid fuels in bioelectrochemical systems^1^,^2^,^3^. Energy recoveries in these devices depend greatly on the electroactivity of the electrode-associated biofilms^4^, yet the mechanism of biofilm conductance is not fully understood. Genetic studies in the model representative *Geobacter sulfurreducens* have identified genes encoding the biofilm exopolysaccharide (EPS) that anchors *c*-type cytochromes to the biofilm matrix^5^. Although the genome of *G. sulfurreducens* contains over 100 genes annotated as *c*-type cytochromes, some of which are required for efficient extracellular electron transfer^6^,^7^,^8^, only one, OmcZ, is essential for biofilm electroactivity^8^,^9^. Other *c*-type cytochromes may be required, but their genetic identification is challenging because of compensatory effects observed when the encoding genes are mutated^7^,^10^.

*In situ* electrical conductivity measurements on living biofilms of *G. sulfurreducens* grown on an electrode demonstrated the thermal dependence of charge transport, which is consistent with the incoherent redox conductivity mediated by the haem groups of the matrix-associated *c*-type cytochromes^11^. The role of cytochromes as electron carriers in the biofilms is further supported by the studies showing their reversible oxidization or reduction as a function of the anode potential^12^,^13^. However, while it is possible to reduce all of the biofilm cytochromes of thin (<10 μm) biofilms with a positive voltage, only half are reduced in thicker (∼20 μm) biofilms^12^. Furthermore, redox potentials decrease sharply as the biofilms grow to or beyond a 20-μm thickness^14^ and a redox gradient is established in these spatial scales that results in the accumulation of reduced cytochrome species further away from the electrode^15^. Source–drain experiments of biofilms grown across 10-μm wide gaps between electrodes indicate that the redox potential already decreases significantly in biofilm regions 10 μm away from the oxidizing electrode^16^. Thus, a redox gradient is established in the biofilms whereby more cytochrome-associated electrons are concentrated in the upper, electron-acceptor-limited biofilm stratum and decrease progressively towards the electrode-attached layers. The redox gradient has been proposed to provide the driving force for electron transport across the biofilms^16^, in a manner analogous to how electrons diffuse through redox polymers hopping among immobilized redox cofactors^17^.

The progressive decline in the rates of cytochrome oxidation and reduction in biofilm layers 10–20 μm away from the electrode indicates that a rate-limiting step exists when biofilms grow to or beyond this thickness. Yet, despite this limitation, *G. sulfurreducens* can grow electroactive biofilms tens of micrometres from the electrode surface while generating current proportionally to the biofilm thickness^18^. Furthermore, cells in the upper biofilm layers are metabolically active and continue to oxidize acetate while contributing to current production^19^. Consistent with this, acetate kinase, which serves as a proxy for acetate oxidative metabolism, is uniformly abundant across the biofilm^20^. By contrast, the expression of the outer membrane *c*-type cytochrome *OmcB* increases fourfold in cells located in the upper stratum^20^, where the redox potential is lowest^14^. This has lead to the proposal that cells in these upper, electron-acceptor-limited zones compensate for the lower rates of cytochrome-mediated redox reactions and maintain optimal rates of respiration by expressing ‘extra electron-transfer machinery'^20^.

One obvious candidate for discharging respiratory electrons and alleviating the electron acceptor limitation of cells in the upper biofilm regions is the conductive pili that *G. sulfurreducens* produces to reduce Fe(III) oxides^21^ and uranium^22^. The pili are also required to grow multilayered biofilms on iron-coated and glass surfaces^23^, plastic surfaces^24^ and on electrodes poised at an oxidizing potential^18^. The need to express pili to form biofilms, and the immunodetection of the outer membrane *c*-type cytochrome OmcS on chemically fixed pili produced by planktonic cells^25^, suggested that the pili may serve as a scaffold for the matrix-associated cytochromes^26^. However, scanning tunnelling microscopy studies did not reveal spectroscopic haem signatures along cell-associated, untreated pili^27^. The significance of OmcS in electroactive biofilms is also questionable, because deletion of the *omcS* gene had no effect in biofilm growth and maximum catalytic current^9^. Furthermore, *omcS* transcripts are less abundant in current-harvesting biofilms than in biofilms grown on the same electrode but operated in open circuit and fed the soluble electron acceptor fumarate^8^. A mere structural role for the pili is also incongruent with *in vitro* conductivity measurements, which show that the pilus fibres are protein nanowires capable of transporting charges at rates several orders of magnitude higher than the rates of cellular respiration^28^. The conductivity measured along individual pilus fibres and thermal dependence of their differential conductance at biologically relevant voltages^28^ is also consistent with the incoherent redox conductivity of living biofilms^11^. Further, a structural model of the pilus fibre refined via molecular dynamics identified transversal and axial multistep charge hopping paths involving aromatic residues (phenylalanines and tyrosines) of the pili required for optimal biofilm electrochemical activity^29^. The pili thus have the attributes necessary to function as biofilm electron carriers and could promote the discharge of respiratory electrons from cells in the electron-acceptor-limited upper regions to the oxidized cytochromes below. This is in accordance with the prediction that electron transfer across electroactive biofilms requires the expression of both cytochromes and pili to promote short-distance, electron-transfer reactions^30^.

Much of the difficulty in assessing the contribution of the pili to biofilm electroactivity stems from the fact that pili-deficient mutants reported thus far also have defects in cytochromes required for extracellular electron transfer^22^,^24^,^31^. Here we investigate the effect of several pili-inactivating mutations in the expression of cytochromes and identify one, a deletion in the gene encoding the pilin polymerization motor PilB, that has no measurable effects in cytochrome expression. Using the *pilB* mutant, we demonstrate that pili expression is required for optimal biofilm electroactivity in thin (∼10 μm) biofilms and to grow the biofilms beyond this threshold distance, which otherwise limits the rates of redox reactions involving the biofilm cytochromes. We also show that the *pilB* defects are similar to Tyr3, a mutant that carries alanine replacements in the three tyrosines of the pilus multistep hopping pathway^29^ and produces pili with reduced conductivity. The results thus indicate that pili, like cytochromes, are electron carriers in anode biofilms, but their ability to discharge respiratory electrons becomes progressively more critical as redox reactions involving cytochromes become rate-limiting in the upper zones. This strategy confers on *Geobacter* cells a metabolic advantage over other bacteria relying solely on cytochromes or diffusible electron carriers, as it maximizes energy generation through extracellular electron transfer even in cells positioned at tens of micrometre distances from the electrode.

## Results

### Inactivation of PilB does not affect cytochrome expression

The assembly of type IVa pili on the bacterial inner membrane is mediated by a protein apparatus involving three interacting subcomplexes: the pilus subcomplex (major pilin and minor pilins, if present), the motor subcomplex (containing several proteins, including the PilB and PilT ATPases that power pilus polymerization and retraction, respectively) and the alignment subcomplex (which properly aligns the assembled pilus through the outer membrane PilQF secretin complex)^32^. As mutations that render the pilus and motor subcomplexes inoperative also prevent pili expression^32^,^33^, we constructed mutants of *G. sulfurreducens* carrying deletions in the genes encoding the PilA pilin subunit (*pilA* mutant) and the PilB ATPase (*pilB* mutant), respectively. In addition, we constructed a pili-deficient mutant (*pilA-E5A*) carrying an alanine substitution in the conserved glutamic E5 amino acid required for proper alignment and assembly of pilins^32^,^34^.

**Figure 1:**
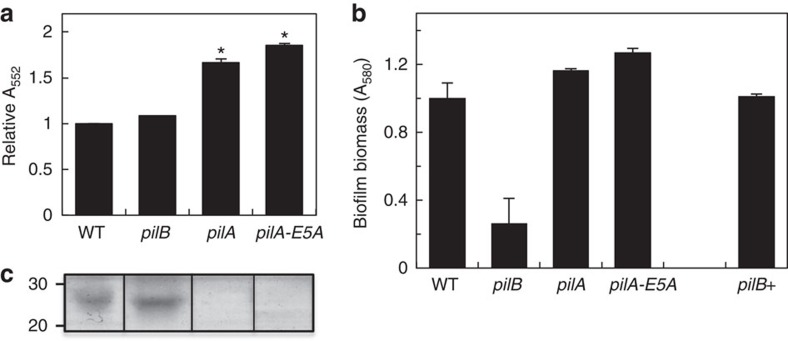
Phenotypic characterization of pili-deficient mutant (*pilB*, *pilA* and *pilA-E5A*) biofilms. (**a**) Relative cytochrome content in biofilm extracts of the pili-deficient mutants in reference to WT (average and s.e. of two biological replicates for each). Significant differences in cytochrome content (*P<0.01) relative to the WT were identified in *t*-test pairwise comparisons. (**b**) Biofilm biomass on plastic surfaces estimated from the OD_580_ of the biofilm-associated crystal violet solubilized with acetic acid (shown are averages and s.d. of six biofilm replicates of the WT, pili-deficient mutants and the genetic complemented *pilB*+ strain). (**c**) OmcZ_S_ band among haem-stained proteins isolated from the biofilm matrix of the WT and pili-deficient strains (full gels are shown in Supplementary Fig. 2c). Lanes were loaded with 20 μg of protein. Numbers at left are relative molecular masses of protein standards in kDa.

Two of the pili-inactivating mutations, *pilA* and *pilA-E5A*, had pleiotropic effects in cytochrome expression. Differential redox spectroscopy of reduced-minus-oxidized ultraviolet–visible spectra (Supplementary Fig. 1) revealed, for example, a greater cytochrome content in the *pilA* and *pilA-E5A* biofilms than in the wild type (WT; Fig. 1a). Similar increases were observed in planktonic cells of the two mutant strains (Supplementary Fig. 2), ruling out any influence of the physiological state of the cells (planktonic or biofilm) in the phenotype. The gene encoding the outer membrane *c*-type cytochrome *OmcB* was also upregulated (Supplementary Fig. 3), suggesting that some of the pleiotropic cytochrome effects observed in the *pilA* and *pilA-E5A* mutants were transcriptional in nature. By contrast, transcript levels for the gene encoding the outer membrane *c*-type cytochrome OmcZ were similar in the WT, and the *pilA* and *pilA-E5A* mutants (Supplementary Fig. 3). Yet, the processed form of this cytochrome (OmcZ_S_, ∼30 kDa), which is released to the biofilm matrix^5^ and is required for optimal biofilm redox activity^8^,^24^, was absent in the matrix of the mutant biofilms (Fig. 1c). OmcZ_S_ was however detected in haem-stained preparations of planktonic cells (Supplementary Fig. 2), suggesting that the inability of the *pilA* and *pilA-E5A* mutant biofilms to secrete this cytochrome was related to the unique physiology of cells living within a surface-attached community.

Pili-deficient mutants have been reported to build thin biofilms on glass surfaces^23^, presumably because the pili provide the structural support needed to stack cells on the surface. Consistent with this, 24-h biofilms of the *pilA* and *pilA-E5A* strains that formed on plastic surfaces were less dense than the WT (Supplementary Fig. 4); however, the mutant biofilms reached WT biofilm biomass levels after 48 h (Fig. 1b; Supplementary Fig. 4). The upregulation of the *xapD* gene, encoding the ATP-dependent exporter of the biofilm EPS^5^, in these mutants suggests that EPS synthesis could have compensated for the pili deficiency during the early stages of biofilm formation, as reported for other bacteria^35^. But once the mutant biofilms reached WT biomass levels at 48 h, the amount of EPS produced per cell was similar to the WT (Supplementary Fig. 5).

In contrast to the pleiotropic nature of the *pilA* and *pilA-E5A* mutations, preventing pilin assembly through the inactivation of the PilB motor (*pilB* mutant) had no measurable effects on the cytochrome content of the biofilms (Fig. 1a). Furthermore, the transcription of genes in the pilin operon (*pilA* and GSU1497) or those encoding the outer membrane *c*-type cytochromes *OmcB* and OmcZ was not affected either (Supplementary Fig. 3). Moreover, OmcZ_S_ was secreted in the *pilB* biofilm matrix (Fig. 1c), and *xapD* transcripts (Supplementary Fig. 3) and biofilm EPS produced per cell (Supplementary Fig. 5) were similar in the *pilB* and WT biofilms. As with other pili-deficient mutants^23^, the *pilB* biofilms that formed on plastic surfaces were less dense than the WT even after 48 h of incubation (Supplementary Fig. 4), as indicated by the reduced biofilm biomass stained with crystal violet (Fig. 1b) and the lower cell protein content extracted from the *pilB* biofilms (75±1% of the WT, average and s.e. of two independent experiments). Yet, the *pilB* biofilm defect was rescued in the genetically complemented *pilB*+ strain, which restored pili production through the expression of the *pilB* gene *in trans* from a medium-copy plasmid (Fig. 1b). Hence, the *pilB* strain provides the elusive genetic tool needed to assess the contribution of *Geobacter* conductive pili in the growth and electrochemical activity of anode biofilms.

### Thin (∼10 μm) biofilms require both cytochromes and pili

**Figure 2:**
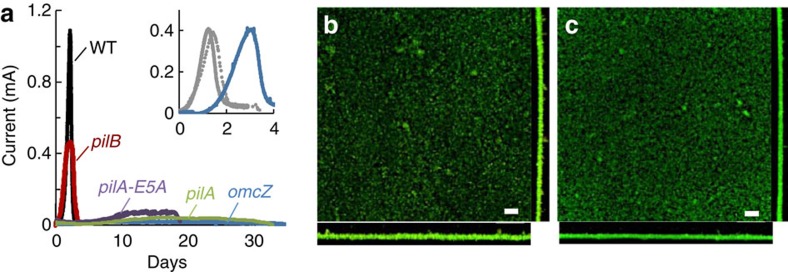
Current production by anode biofilms of WT and mutants in MECs fed with 1 mM acetate. (**a**) Representative plots of current generation by WT, pili-deficient strains (*pilB*, *pilA* and *pilA-E5A*) and *omcZ* mutant in MECs operated in batch and fed with an initial concentration of 1 mM acetate. Inset shows current generation (*Y* axis, in mA) over time (*X* axis, in days) by double mutants *pilB mshE* (solid grey), *pilB gspE* (dashed grey) and an equal mixture of *pilB* and *omcZ* cells (blue). (**b**,**c**) Confocal micrographs showing top and side views of anode biofilms of the WT (**b**) and *pilB* (**c**) strains collected at the end of the MEC experiment, when current had decreased to <0.1 mA, and stained with the BacLight viability kit (green, live; red, dead). Scale bar, 20 μm.

We investigated the ability of the pili-deficient mutants to generate current in microbial electrolysis cells (MECs) fed an initial concentration of 1 mM acetate and operated in batch in reference to the WT strain. Under these conditions, all of the strains tested grew reproducibly to a thickness of ca. 10 μm, but the rates of current production and the maximum current reached were reduced in the three pili-deficient mutants (Fig. 2a). The *pilA* and *pilA-E5A* mutant biofilms, which in addition to the pili deficiency do not secrete OmcZ_S_ in the biofilm matrix (Fig. 1c), reached low levels of current (0.08±0.05 and 0.14±0.08 mA, respectively). As a result, prolonged incubations were required for the mutant biofilms to oxidize all the acetate in the anode chamber (Fig. 2a) and to reach WT thickness (∼ 13.5±3.5 and 11.6±1.9 μm, respectively, in duplicate MECs). Similar low levels of current (0.11±0.07 mA for duplicate MECs) and lengthy incubation times were recorded in control MECs driven by a null *omcZ* mutant (Fig. 2a), consistent with the need of the biofilms to concentrate OmcZ_S_ near and on the electrode for efficient electron transport to the anode surface^36^. The expression of OmcZ_S_ in the *pilB* biofilm matrix (Fig. 1c) improved MEC performance compared with the *pilA* and *pilA-E5A* mutants, but did not fully restore WT levels of current (Fig. 2a). Maximum current harnessed from the *pilB* anode biofilms in triplicate MECs averaged, for example, 0.52 (±0.06 mA), which is about half of the maximum current produced by WT biofilms (1.06±0.05 mA).

The partial restoration of biofilm electroactivity in *pilB* compared with the other pili-deficient mutants did not result from functional complementation by the PilB homologues MshE and GspE (putative ATPase motors of type II secretion systems) because deleting the *pilB* and *mshE* or *gspE* genes generated double mutants that were phenotypically indistinguishable from the *pilB* single mutant (Fig. 2a, inset). In addition, the *pilB* mutant defect could not be complemented in MECs co-inoculated with the *pilB* and *omcZ* mutant cells (Fig. 2a, inset). We did observe a delay in current generation in the co-culture-driven MECs, which is the phase when cells colonize the electrode before growing on it while coupling the oxidation of acetate to the reduction of the electrode^37^. However, once current production was initiated, the performance of the co-culture MECs was similar to the *pilB* MECs. This suggests that biofilm cells need to express both pili and OmcZ_S_ for optimal biofilm growth and electroactivity.

The rates of current increase in the *pilB* MECs, which correlate with the exponential phase of biofilm growth^37^, were also reduced in half (0.61±0.15 mA per day) compared with the WT biofilms (1.45±0.06 mA per day). However, coulombic efficiencies in the *pilB*-driven MECs (96.6±3.1) were similar to the WT (94.6±4.1), indicating that, on average, the *pilB* biofilms converted the same amount of acetate to electricity as the WT biofilms, they just did so at slower rates. Confocal micrographs of WT and *pilB* biofilms collected at the end of the experiment, when all of the acetate had been depleted, and stained with fluorescent viability dyes showed predominantly live cells and similar biofilm structure (Fig. 2b,c, respectively). Furthermore, the biofilm biomass estimated from the fluorescence emitted by the biofilm cells was similar in both strains (10.05±1.00 in WT and 9.06±1.46 μm^3^ μm^−2^ in *pilB*). Thus, the *pilB* defects cannot be attributed to a reduction in the number of cells actively contributing to electron transfer or structural variations of the biofilms. Furthermore, the *pilB* phenotypes in MECs fed with 1 mM acetate (Fig. 2) are similar to those reported for Tyr3, a mutant that produces pili with alanine replacements in the pilin's three tyrosines of the pilus multistep hopping pathway that impair the cell's ability to transfer electrons to extracellular electron acceptors such as Fe(III) oxides^29^. Hence, the *pilB* phenotypes are consistent with reductions in the respiratory rate of individual cells resulting from the inability of the mutant cells to discharge electrons using the pili.

### The conductivity of the pili is required to grow thick biofilms

**Figure 3:**
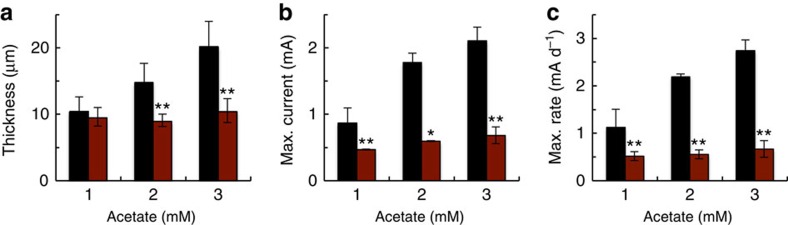
Thickness and electroactivity of WT and *pilB* biofilms. Anode biofilm thickness (**a**), maximum current (**b**) and maximum rates of current production (**c**) in batch MECs fed with an initial concentration of 1, 2 and 3 mM acetate and driven by the WT (black) or the *pilB* mutant (maroon) strains. Shown are average and s.d.'s or errors of four (1 mM) or two (2 and 3 mM) independent MEC experiments, respectively. Significant changes between the WT and *pilB* values at each acetate concentration are indicated with stars (*P <0.05; **P<0.005). Representative plots of current generation used to estimate biofilm thickness and electroactivity at each acetate concentration are shown in Supplementary Fig. 6.

By increasing the initial amount of electron donor (acetate) added to the anode chamber of the MECs operated in batch, we were able to increase the thickness of the WT anode biofilms from ∼10 to 15 μm (2 mM acetate) and 20 μm (3 mM acetate; Fig. 3a). These are spatial scales that lead to the progressive accumulation of reduced cytochromes in the upper biofilm layers, thus limiting the ability of the cells to use cytochromes in these regions as electron acceptors for respiration^12^. Yet, cells in these electron-acceptor-limited layers are actively respiring and contributing to current production. To investigate a potential role for the conductive pili in alleviating the limitations imposed by the reductions of redox potential in the upper biofilm stratum, we grew the *pilB* mutant in MECs fed with 2 and 3 mM acetate. In contrast to the linear increases in WT biofilm thickness with initial acetate feeding, the *pilB* biofilms remained thin (∼10 μm) under all the conditions tested (Fig. 3a). Similarly, parameters that measure the biofilm electroactivity such as current maxima and rates of current production increased proportionally to acetate availability in the WT MECs, but were unaffected in the *pilB* MECs (Fig. 3b,c).

**Figure 4:**
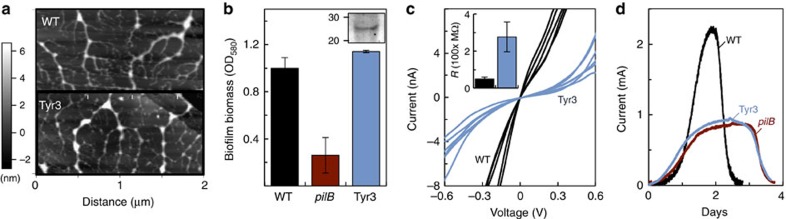
Role of pilus conductivity in the growth and electroactivity of anode biofilms. (**a**) AFM topographic images of pili purified from the WT and Tyr3 strains and deposited on a HOPG surface. (**b**) Crystal-violet-stained biomass of 48-h-old biofilms of the *pilB* and Tyr3 mutants relative to WT (shown are averages and s.d.'s of six biofilm replicates for each strain). Inset: OmcZ_S_ band in haem-stained proteins isolated from the biofilm matrix of the Tyr3 biofilms (20 μg of protein loaded per lane; full gel is shown in Supplementary Fig. 2d). Numbers at left are relative molecular masses of protein standards in kDa. (**c**) Representative current–voltage (*I*–*V*) plots obtained after probing the transversal conductivity of individual WT and Tyr3 pili (**a**). Inset shows the average and s.d. of the electrical resistance of four or more WT or Tyr3 pili, each probed in at least three positions. (**d**) Current production by WT, *pilB* and Tyr3 biofilms grown in MECs fed initially with 3 mM acetate.

As a control, we also characterized the Tyr3 mutant strain phenotypically (Fig. 4). The Tyr3 mutant produced pili at WT levels (Fig. 4a), which provided the structural support needed to grow dense biofilms on plastic surfaces when fumarate was provided as the electron acceptor (Fig. 4b). The Tyr3 biofilms also expressed OmcZ_S_ in the biofilm matrix (Fig. 4b, inset). However, as predicted in molecular dynamics simulations^29^, the reduced number of aromatic contacts in the Tyr3 pili increased their electrical resistance (∼280 MΩ) fivefold over that measured in the WT pili (∼50 MΩ) (Fig. 4c). Furthermore, Tyr3 anode biofilms in MECs fed with 3 mM acetate remained thin (13.3±2.4 μm, for duplicate MECs) and reached current levels (0.81±0.10 mA, for duplicate MECs) comparable to those recorded in the *pilB*-driven MECs and fed the same amount of acetate (Fig. 4d). Thus, the ability of cells to grow thick (>10 μm) biofilms on the anode electrode while coupling the oxidation of acetate to current generation depends on the conductivity, rather than the structural support, provided by the pili.

## Discussion

The studies described herein provide the elusive genetic evidence, in the form of the *pilB* mutant, needed to assess the contribution of the conductive pili of *G. sulfurreducens* to the electrochemical activity of electrode-associated biofilms. Indeed, the *pilB* mutant had no measurable changes in *c*-type cytochromes (Fig. 1) and was able to form biofilms of WT thickness (∼10 μm) in MECs operated in batch and fed an initial acetate concentration of 1 mM (Fig. 2). Yet, despite forming anode biofilms as thick as the WT under these conditions, the pili deficiency prevented the mutant from generating current at the same rates as the piliated WT strain and reduced current maxima (Fig. 3). Furthermore, the *pilB* mutant was unable to grow biofilms beyond this threshold thickness when the initial concentration of acetate fed to the MECs was increased from 1 to 2 or 3 mM, which are conditions that allow the WT biofilms to grow ∼15- and 20-μm thick, respectively (Fig. 3). We can rule out mass transport limitations because the biofilms investigated in this study were too thin (<50 μm) to restrict the diffusion of the electron donor, acetate^38^. Furthermore, the phenotypes of the pili-deficient *pilB* mutant in MECs were similar to those of a Tyr3 mutant, which produces pili with reduced conductivity (Fig. 4). This finding challenges the proposal that the pili's role is restricted to providing structural support needed for cytochrome organization^26^ and is instead consistent with the need of the biofilm cells to express conductive pili to transport electrons.

The genetic evidence therefore supports a model in which the conductive pili and the cytochromes work coordinately as electron carriers to maintain optimal rates of electron transfer in thin (∼10-μm thick) biofilms (Fig. 2). This is in accordance with the early models that suggested that electron transfer across electroactive biofilms may require the expression of both cytochromes and pili to promote short-distance, electron-transfer reactions^30^. However, as the biofilms grow further away from the electrode, the cytochromes become progressively more reduced and the ability of the pili to discharge respiratory electrons becomes more critical (Fig. 3). The redox potential decreases significantly already in regions of the biofilm only 10 μm away from an oxidizing electrode^16^. When compared with a 10-μm thick WT biofilm, the electrochemical activity of the *pilB* biofilms (rates of current production and maximum current) was reduced approximately in half (Figs 2 and 3). The *pilB* defects cannot be attributed to decreases in the number of biofilm cells contributing to electron transfer, because the biofilm thickness and the number of viable cells in the *pilB* biofilms were comparable to the WT. EPS production (Supplementary Fig. 5) and OmcZ_S_ expression in the biofilm matrix (Fig. 1c) were also similar in the *pilB* and WT biofilms. The reductions in electrochemical activity of 10-μm *pilB* biofilms are also within the ranges reported for Tyr3 biofilms grown to a similar thickness^29^. Tyr3 pili are expressed at WT levels, but carry alanine replacements in the three tyrosines of the pilus multistep hopping pathway^29^ that reduce the conductivity of the pili more than fivefold (Fig. 4). We measured, for example, an average electrical resistance of∼50 MΩ for WT pili deposited on highly oriented pyrolytic graphite (HOPG), which is within the ranges reported for pili deposited on gold electrodes^28^, and ∼280 MΩ in the Tyr3 pili. Thus, the reduced electrochemical activity of the *pilB* and Tyr3 biofilms results from the inability of the mutant cells to discharge respiratory electrons via the pili.

*In vitro* charge transport measurements indicate that individual pilus fibres can transport charges at micrometre distances and at rates (ca. 10^9^ electrons per second for a 1-μm-long pilus at 100 mV) that greatly exceed the cellular respiratory rates^28^. Furthermore, the pili can reach micrometre lengths^21^ and physically interact with extracellular cytochromes^25^ of the biofilm matrix^5^. This could allow the biofilm pili to directly discharge electrons from the cells to matrix-associated cytochromes such as OmcZ_S_. This multihaeme *c*-type cytochrome preferentially localizes closer to the electrode and on its surface^36^ and has the wide redox potential working range^39^ needed to function as an electrochemical gate between the biofilm and the electrode^36^. Indeed, pilin mutations that resulted in defects in OmcZ_S_ expression (*pilA* and *pilA-E5A*; Fig. 1) reduced the capacity of the biofilms to produce current to levels similar to a control *omcZ* mutant (Fig. 2). The *pilA* and *pilA-E5A* mutant cells also grew very slowly on the anode, taking several days to oxidize the acetate and reach WT biofilm thickness (∼10 μm). The expression of OmcZ_S_ in the *pilB* biofilm matrix (Fig. 1c) alleviated the defect but only partially (Fig. 2). Thus, the pili are required for optimal current production within spatial scales (∼10 μm) where biofilm cytochromes, including OmcZ_S_, are available as electron carriers.

The coordinated interaction between pili and cytochrome electron carriers in the biofilm layers closer to the electrode is further supported by the cross-regulation of pili assembly and cytochrome export observed in this and one other study^40^. Earlier studies demonstrated that the PilA pilin protein of *G. sulfurreducens* is translated as both a short and a long prepilin isoform, which interact to promote the processing and assembly of the short pilin and the export of *c*-type cytochromes such as OmcZ to the outer membrane^40^. Indeed, we showed that mutations that prevented pilin expression or disrupted pilin–pilin interactions (*pilA* and *pilA-E5A*, respectively) interfered with the pilin's role in secretion and lead to defects in cytochrome expression and localization (Fig. 1). Similarly, *pilA*-deficient mutants used in earlier genetic studies^18^,^21^,^41^ also had defects in *c*-type cytochromes required for optimal extracellular electron transfer^22^,^24^. The dual role that *Geobacter* pilins play in pili synthesis and cytochrome export is in accordance with the multiple biological roles reported for type IV pilins from other bacteria. In *Pseudomonas aeruginosa*, for example, the structural subunit of the pilus fibre (the PilA pilin) interacts with components of the general secretion pathway to promote the export of proteins across the outer membrane^42^. As a result, mutations that inactivate PilA in this bacterium affect the secretion of numerous proteins and produce mutant strains with multiple phenotypes. By contrast, the function of PilB is restricted to pilus biogenesis. Thus, inactivating mutations in the *pilB* gene of *P. aeruginosa* prevent pili expression, but do not cause export defects^43^. Similarly, inactivating the PilB motor of *G. sulfurreducens* prevents the assembly of the pilins, but does not interfere with pilin interactions needed for cytochrome export. As a result, cytochrome expression in the *pilB* mutant was as in the WT strain (Fig. 1).

As the biofilms grow further apart from the electrode (beyond a threshold distance of ∼10 μm), the reversible oxidization or reduction of biofilm cytochromes becomes rate-limiting^12^. Redox potentials decrease sharply beyond this threshold distance^14^,^16^ and reduced species progressively accumulate in biofilm layers further away from the electrode^15^. The redox gradient provides the driving force for electron transport across the biofilms^16^, but also limits the ability of cells in the upper layers to discharge respiratory electrons to nearby cytochromes. It has been proposed that cells in these outer regions of the biofilm overcome this limitation by upregulating the expression of electron transport proteins involved in extracellular electron transfer^20^. Consistent with this model, while it was possible to grow thicker (ca. 15 and 20 μm) WT biofilms in MECs, preventing the assembly of the pilins (*pilB* mutant) or reducing the conductivity of the assembled pili (Tyr3 mutant) effectively prevented the biofilms from growing >10 μm away from the electrode (Fig. 4). Hence, the expression and conductive properties of the pili are required to maintain optimal electrical connectivity as the biofilms grow in thickness and the cytochromes become progressively more reduced. These upper layers of the biofilm may be electron acceptor limited, but are also the regions where electron donor (acetate) availability is highest^19^. Studies show that *G. sulfurreducens* can continue to metabolize acetate in the absence of an electron acceptor by overexpressing its extracytoplasmic cytochromes and using them as a capacitor to store up to 10^6^ electrons per cell^44^. Indeed, the outer membrane *c*-type cytochrome *OmcB*, which is transcriptionally upregulated under conditions of electron acceptor limitation^45^, is four times more abundant in cells from the 10–20-μm upper biofilm stratum than in the biofilm layers closer to the electrode^20^. This suggests that cells in these upper biofilm regions could continue to metabolize acetate and generate a proton motive force for energy generation, accumulating electrons in the cytochromes of the cell envelope. The pilus apparatus is anchored in the cell envelope of Gram-negative bacteria; hence, it could potentially accept electrons from the extracytoplasmic cytochromes, promoting their discharge from the cell to the biofilm matrix. By growing several micrometres in length^21^ and intertwining to form a complex web of nanowires^27^, the pili could electronically connect the upper and bottom strata of the biofilm, effectively bypassing the rate limitation imposed by the accumulation of reduced cytochromes in the electron-acceptor-limited regions.

**Figure 5:**
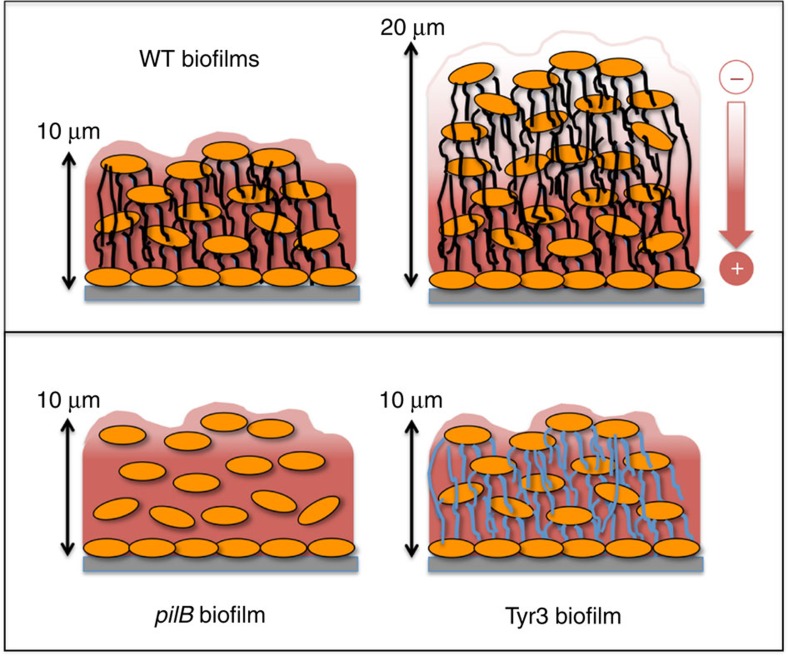
Model of mechanistic stratification of electroactive biofilms. The pili permeate the biofilm matrix and promote the discharge of respiratory electrons to the oxidized cytochromes (in red, positive charge) in thin (∼10-μm thick) biofilms. As the biofilms grow in thickness, reduced cytochromes accumulate (in white, negative charge) and the pili provide the means for the cells to discharge respiratory electrons to the oxidized cytochromes below. In the absence of pili (*pilB* biofilms) or in biofilms expressing poorly conductive pili (Tyr3 biofilms), biofilm growth is limited to the thickness (∼10 μm), where oxidized cytochromes are available to serve as electron acceptor to the cells.

Taken together, the results support a model of mechanistic stratification in electroactive biofilms mediated by the pilus nanowires (Fig. 5). According to the model, the conductive pili network works coordinately with the matrix-associated cytochrome carriers to transfer electrons in the region (<10 μm) closer to the electrode. However, the pili become the primary mechanism for discharging respiratory electrons in the upper (>10 μm) region, where cytochromes are predominantly in a reduced state (Fig. 5). The pili can discharge electrons at micrometre distances and at rates that greatly exceed the cellular respiratory rates^28^. These attributes could allow the pili network to electronically connect cells in the upper layers of the biofilm to the oxidized cytochromes present in the regions closer to the electrode. As a result, mutants that do not assemble (*pilB*) or produce poorly conductive (Tyr3) pili cannot grow beyond this threshold distance and have reduced electroactivity compared with WT biofilms of comparable thickness (Fig. 5). The coordinated interactions between pili and cytochrome electron carriers revealed in our genetic studies challenge the proposal^41^ that metallic-like pilus conductivity fully accounts for electron flow across the biofilms and is in accordance with the prediction^30^ that electron transfer across electroactive biofilms involves short-distance, multistep hopping reactions between pili and cytochromes. Furthermore, living biofilms show the thermal dependence of incoherent conductivity predicted for electron-transfer reactions involving haems of *c*-type cytochromes^11^ and also demonstrated for charge transport via pili^28^. Phenylalanine and tyrosine residues cluster during pilin assembly, creating axial and transversal pathways with inter-aromatic distances and dimer geometries optimal for multistep hopping^29^. The tyrosine residues are in close proximity to acidic residues, which could be transiently protonated to promote proton-coupled electron transfer between tyrosine residues^29^. The simultaneous transfer of a proton and an electron lowers the oxidation potential of the tyrosine residue to the levels needed to enable fast rates of electron transfer in biological systems^46^,^47^. The transient protonation of acidic residues of the pili during charge transport also makes the rates of charge transport along the pili pH dependent. Thus, pH gradients that establish across anode biofilms^14^,^48^ likely tune the rates of charge transport via the pili and interactions with cytochromes, particularly in the biofilm regions closer to the electrode, where the pH is lower.

In addition to pH gradients, redox and electrical gradients form across the biofilms that can influence the physiology of the cells and could control the efficiency of charge transport along the pili and interactions with cytochromes to maintain optimal rates of electron transfer. Nutrient gradients also form in biofilms, which can influence the cell physiology and, therefore, the expression of biofilm electron carriers^13^,^14^. Microarray analyses of thin sections of anode biofilms of *G. sulfurreducens* did not detect changes in the expression of the *pilA* gene^49^. However, *pilA* is also transcribed under conditions that do not result in pili assembly^50^. Thus, the levels of cell piliation across the biofilms could be modulated by the local biofilm microenvironment to maintain optimal rates of respiration, as observed for other respiratory components^20^. The regulatory networks that control the expression and electrochemical activity of electron carriers such as the pili may be responsive to more than one biofilm parameter, such as pH, nutrient availability and redox potential. Biofilms of oxygen-respiring bacteria are, for example, responsive to redox potentials and use redox cues from their microenvironment to alter the biofilm structure, maximize oxygen diffusion and maintain redox homeostasis^51^. Similar mechanisms could control the expression of pili and cytochromes to facilitate their coordinated interactions and redox homeostasis in *G. sulfurreducens* biofilms. Thus, understanding the multiple functions that pili play in *G. sulfurreducens* and their regulation may prove instrumental to improve the performance of *Geobacter*-driven electrochemical systems for applications in bioenergy.

## Methods

### Bacterial strains and culture conditions

The bacterial strains used in this study are described in Supplementary Table 1. The WT strain, *G. sulfurreducens* strain PCA (ATCC 51573), was kindly provided by Daniel Bond (University of Minnesota) and was used to construct three knock-out mutants: *pilB* (carrying a deletion in the *pilB* gene, GSU1491), *pilA* (carrying a deletion in the pilin gene *pilA*, GSU1496) and *omcZ* (carrying a deletion in the *omcZ* gene, GSU2076). When indicated, the *pilB* mutation was complemented *in trans* by expressing a WT copy of *pilB* from the pRG5 plasmid (*pilB*+ strain). The WT strain was also used to construct the *pilA-E5A* mutant (carrying a single alanine replacement of the glutamic 5 residue in the mature pilin PilA protein) and the Tyr3 mutant (carrying alanine replacements in the pilin's three tyrosine residues Y27, Y32 and Y57). The WT and mutant strains were routinely cultured anaerobically in NB medium^52^ supplemented with 15 mM acetate as the electron donor and 40 mM fumarate as the electron acceptor (NBAF) and 0.1% yeast extract and 1 mM cysteine (NBAFYE). For biofilm assays, cells were cultured in modified freshwater FW medium^22^ with 30 mM acetate and 40 mM fumarate. Cells used to inoculate the anode chamber of MECs were grown in mineral DB medium^53^ supplemented with 20 mM acetate and 40 mM fumarate (DBAF).

### DNA manipulations and mutant construction

Deletions of the *pilB*, *pilA* and *omcZ* genes were constructed using the *cre–lox* system^54^ with the primers listed in Supplementary Table 2. The general procedure included the PCR amplification of the gentamycin (Gm) resistance cassette (*aaaC1*) flanked by *loxP* sites (Gm-*loxP*) from plasmid pCM351 (ref. 54) using primer set RS21–RS22 and of the upstream/downstream target chromosomal regions using primer sets RS1–RS2/RS3–RS4 (*pilB*), RS5–RS6/RS7–RS8 (*pilA*) and RS9–RS10/RS11–RS12 (*omcZ*). The upstream region of the target gene, the Gm-*loxP* cassette and the downstream region of each gene were then fused in that order by overlap extension PCR using the corresponding external forward and reverse primers, and the Herculase II Fusion DNA Polymerase (Agilent Technologies). PCR conditions for this last amplification step were: 2 min of denaturation at 95 °C; 35 cycles of 20 s at 95 °C, 25 s at 54 °C and 2 min at 72 °C; and a final 3-min extension at 72 °C. The PCR products were then separated on an agarose gel, purified using the Zymoclean Gel DNA Recovery kit (Zymo Research), and cloned into the pCR2.1 plasmid (Supplementary Table 2) using the TOPO TA Cloning kit (Life Technologies) for sequence confirmation. After cloning, the constructs were PCR-amplified with the external primers, purified from an agarose gel and electroporated into electrocompetent cells of *G. sulfurreducens* following a previously published procedure^52^. Selection of recombinant strains was performed on NBAFYE plates supplemented with 5 μg ml^−1^ of gentamycin. When indicated, the Gm cassette carried by the *pilB* mutant was excised from its chromosomal location by expressing the Cre recombinase from plasmid pCM158 (ref. 54) and selecting for transformants on NBAFYE plates supplemented with 200 μg ml^−1^ of kanamycin. The excision of the marker was confirmed by PCR and the resulting mutant (*pilB*) was transferred twice in NBAFYE medium without kanamycin and then plated on NBAFYE with and without kanamycin to confirm the loss of the plasmid. The *pilB* strain was used to construct the double *pilB gspE* and *pilB mshE* mutants (Supplementary Table 1) using the general PCR procedure described above but with primer sets RS9–RS12, RS13–RS16 and RS17–RS20 (Supplementary Table 2), respectively.

We constructed the *pilA-E5A* and Tyr3 mutants by introducing one (E5A) or three (Y27A, Y32A and Y57A) alanine replacements, respectively, in targeted codons in the *pilA* gene (GSU1496). The primers used to construct these mutants are listed in Supplementary Table 2. The general procedure was to PCR amplify the *pilA* gene with its upstream chromosomal region (primers RS23–RS24), the downstream region of *pilA* (primers RS25–RS26) and a spectinomycin cassette (Sp; *aadA* gene) from plasmid pRG5 (ref. 10; primers RS27–RS28). The three fragments were then fused by overlap extension PCR (primers RS23 and RS26) to generate a 1684-bp DNA construct (*pilA*-Sp) containing the *pilA* gene and its upstream region, the antibiotic cassette (*aadA*), and the *pilA* downstream region. The construct was gel-purified and cloned into plasmid pCR2.1-TOPO TA vector (Invitrogen). Targeted nucleotide substitutions in the *pilA* gene were introduced using the QuikChange Lightning Site-Directed Mutagenesis kit (Agilent Technologies) using primers RS29 and RS30 (E5A substitution). Tyr3 was generated by sequentially introducing Y27A (RS31–RS32), Y32A (RS33–RS34) and, last, Y57A (RS35–RS36), as previously described^29^. The mutated fragments were confirmed by sequencing before PCR amplification using the external primers, gel purification and electroporation into electrocompetent cells of *G. sulfurreducens*. Selection of recombinant strains was performed on NBAFYE plates supplemented with 75 μg ml^−1^ of spectinomycin.

### Reverse Transcription-quantitative–PCR

Transcripts for key components of the biofilm matrix (pili, EPS matrix and matrix-associated *c*-type cytochromes) were quantified by reverse transcription-quantitative PCR (RT–qPCR). The gene targets were the pilin-encoding gene *pilA* (GSU1496) and its downstream gene (GSU1497) in the *pilA* operon^21^, the ATP-dependent EPS exporter *xapD* (GSU1501)^5^, the outer membrane *c*-type cytochrome *omcB* (GSU2737) (ref. 6) and *omcZ* (GSU2078), which encodes for the precursor of the matrix-associated *c*-type cytochrome OmcZ_S_ (ref. 39). The constitutive gene *rpoD* (GSU3089) was used as a control. WT, *pilB*, *pilA* and *pilA-E5A* biofilms were grown for 48 h in the wells of 48-well polystyrene plates containing 600 μl of FWAF before decanting the culture broth and resuspending the biofilm cells in 50% (v/v) of ice-cold methanol to stop transcription. The cell suspension was then centrifuged to isolate the cells as a pellet and trizol reagent (Invitrogen) was used to extract their RNA. RNA treatment with RNase-free DNase (Promega) and reverse transcription with random primers (Promega) using the Super Script III Reverse Transcriptase (Invitrogen) were carried out following manufacturers recommendations. RT–qPCR was performed using the rEVAluation qPCR Master Mix (Syzygy), as recommended by the manufacturer, using the primers listed in Supplementary Table 2. The comparative C_T_ method^55^ was used to calculate the relative expression of each gene using *rpoD* constitutive expression as an internal control (ΔC_T_ value or C_T (target)_−C_T (rpoD)_) for each strain, and the average of the difference between each mutant strain ΔC_T_ and the WT ΔC_T_ was used to calculate the ΔΔC_T_. The relative fold change of expression for each target gene versus the *rpoD* internal control for each mutant strain versus the WT was then calculated with the formula 2^−ΔΔCT^ for four replicate samples. Statistically significant changes in gene expression relative to *pilB* were determined in pairwise comparisons using the *t*-test function of the Microsoft Excel software.

### Microbial electrolysis cells

The growth and electrochemical activity of the WT and mutant biofilms were assayed in H-type MECs equipped with anode and cathode graphite rod electrodes (Alfa Aesar, 1.27-cm diameter, 99% metals basis, 12 cm^2^) and a 3 M Ag/AgCl reference electrode (Bioanalytical Systems, Inc.). The MECs were set up, inoculated with cell suspensions harvested from early stationary phase DBAF cultures, filled with DB medium and operated in batch with a poised anode electrode (0.24 V versus reference electrode) as described previously^53^, except that the electron donor in the anode chambers was acetate, provided in 1, 2 or 3 mM concentrations, as indicated. Supernatant samples were periodically removed from the anode medium broth, filtered (0.45 μm) and analysed by high-performance liquid chromatography (HPLC), as described elsewhere^56^, to monitor electron donor removal. Current production was recorded with a VSP potentiostat (BioLogic) and biofilm electroactivity was inferred from the rates of linear current production (mA per day) before reaching maximum current and initiation of the deceleration phase. The exponential phase of current production was also fitted statistically (*R*^2^>0.98) to an exponential curve and the exponent of the resulting formula was used to estimate the generation times of the biofilm cells growing exponentially on the anode electrode.

At the end of the MEC experiment, when current had decreased to <0.1 mA, the anode electrodes were removed and the live and dead biofilm cells were differentially stained in green and red, respectively, with the SYTO-9 and propidium iodide dyes of the BacLight viability kit (Invitrogen). The anode electrodes with the stained biofilms were then immersed gently in a Lab-Tek coverglass chamber (Nunc) filled with 3 ml of PBS and examined using a FluoView FV1000 inverted microscope system (Olympus, Center Valley, PA) equipped with an Olympus UPLFLN × 40 oil immersion objective (numerical aperture, 1.30). SYTO-9 was excited at 488 nm and propidium iodide was excited at 543 nm. Vertical two-dimensional images of the biofilms were collected every 1 μm from ∼10 random fields (1,024 by 1,024 pixels, 0.31 μm per pixel) per electrode, using a minimum of two biological MEC replicates. The biofilm thickness was then manually analysed by averaging thickness measurements of five representative areas per field. When indicated, the COMSTAT software^57^ was used to estimate other biofilm structural parameters, as described previously^53^.

### Static biofilm assays on plastic surfaces

The WT and mutant strains were also grown on plastic surfaces using a soluble electron acceptor (fumarate), essentially as described elsewhere^24^. Briefly, the strains were grown in FWAF to mid-exponential phase and inoculated to a final OD_600_ of 0.02 in 150 μl of FWAF dispensed in each of six replicate wells of a 96-well, polystyrene, tissue culture-treated plate (Costar, Corning Life Sciences). Unless otherwise indicated, the plates were incubated for 48 h at 30 °C and the optical density of the culture at 600 nm (OD_600_) measured before discarding the culture broth and staining the biofilms for 30 min with 150 μl of a 0.01% (w/v) aqueous solution of crystal violet. After staining, the biofilms were washed with double-distilled H_2_O and dried overnight before resolubilizing the biofilm-associated crystal violet with 33% (v/v) acetic acid. The optical density of the solution at 580 nm (OD_580_) was used to estimate the biofilm biomass (cells and matrix) and the biofilm biomass for each strain was averaged for six replicate samples. Time-course biofilm assays were performed the same way and biofilm growth was expressed as the biofilm biomass that stained with crystal violet (OD_580_) relative to the OD_600_ of the planktonic culture.

When indicated, 48-h WT and *pilB* biofilms grown in the wells of three 48-well plates were collected and treated with SDS, as described earlier^24^, to lyse the cells and solubilize the total biofilm protein. Protein in the biofilm lysates was estimated with the Pierce BCA protein assay (Thermo Fisher Scientific). We estimated that ∼90% of the protein solubilized with SDS was contributed by the biofilm cells, thus serving as a proxy of cell numbers in the biofilms.

### Isolation and characterization of the biofilm matrix

The WT and mutant strains were each grown in the wells of two 48-well plates for 48 h to grow biofilms, as described above. The biofilm biomass from the two 48-well plates was then scraped and pooled together, and biofilm lysates were generated by SDS treatment, as described above for the isolation of cell protein. The biofilm matrix was isolated as previously described^24^, except that the biofilm lysate was generated by repeated passages of the biofilm biomass through an 18-G needle and included several steps of centrifugation and washes to isolate the insoluble biofilm matrix fraction. The sample was lyophilized and its EPS component was acid hydrolysed, as previously described^1^. EPS content was calculated as glucose equivalents using a published protocol^5^, except that the glucose content in the acid-hydrolysed sample was estimated by HPLC, using a Waters HPLC instrument (Milford, MA) equipped with the refractive index and ultraviolet detectors, and operated as described elsewhere^58^.

### Assays for cytochrome content and profiling

The cytochrome content of 48-h biofilms and planktonic cells of the WT and mutant strains was estimated by redox difference spectroscopy. The biofilms were first grown for 48 h in 96-well plates, as described above for the biofilm assays on plastic surfaces, before discarding the culture broth and storing the plates at −80 °C for a minimum of 24 h to freeze the biofilms. Before redox difference spectroscopy, the plates with biofilms were thawed at room temperature for a minimum of 15 min before suspending the biofilms in FW medium. A total of 96 biofilm samples (that is, 96 wells) were pooled together for each strain and the OD_600_ of the biofilm cell suspension was adjusted to 0.2 with FW medium. SDS (final concentration of 0.1% w/v) was then added to the cell suspension to lyse the cells and fresh 1 mM dithionite was added to the cell extract as a reducing agent anaerobically inside a glove bag (Coy Laboratories). The ultraviolet–visible spectrum from 350 to 650 nm of the reduced cell extract was then collected before and after oxidation with 2 mM ferricyanide using a Shimadzu UV-2401 spectrophotometer. The difference between the reduced and oxidized spectra at 552 nm (the α-Soret band in the reduced state) of the biofilm cell extracts in the WT and mutants (*pilB*, *pilA* and *pilA-E5A*) was used to estimate the overall biofilm cytochrome content (cells and biofilm matrix)^59^. Spectra were also collected from cell extracts obtained from planktonic cells collected from exponential (OD_600_. 0.4–0.5) cultures grown in FWAF at 30 °C. The total haem content of the cells was analysed using the alkaline pyridine haemochrome method^60^. Statistically significant changes in cytochrome content relative to the WT were determined in pairwise comparisons using the *t*-test function of the Microsoft Excel software.

Cytochromes were also extracted with SDS from the biofilm matrix, isolated as described above and separated electrophoretically by SDS–polyacrylamide gel electrophoresis in 12% Mini-Protean TGX gels (Bio-Rad). Novex Sharp molecular-weight markers (Invitrogen) were used as standards. Haem-containing protein bands were stained with 3,3′,5,5′-tetramethylbenzidine, as previously described^61^. Replicate gels were run and stained with Coomassie blue to ensure that the total protein content per lane was comparable. When indicated, SDS–polyacrylamide gel electrophoresis and haem staining were also used to profile all haem-containing proteins within planktonic cells grown in FWAF to mid-exponential phase.

### Pili purification and conductivity measurements by CP-AFM

Pili from WT or the Tyr3 mutant strain of *G. sulfurreducens* were purified as previously described^22^, except that all buffers contained 1 mM EDTA and all drying steps were carried out under a constant flow of filter-sterilized (0.22 μm) N_2_ gas. The pili were deposited on the surface of freshly cleaved HOPG for 30 min, then blotted dry, to probe the transversal conductivity of hydrated pili by conductive probe-atomic force microscopy (CP-AFM). The sample was scanned with the AFM tip in tapping mode to image the pili and to identify individual fibres. The CP-AFM tip was then positioned at different points along each pilus filament to measure its transversal conductivity while applying a bias voltage within the ±1 V range (3 nN force, 1 Hz rate). To ensure reproducibility, two to three current–voltage (*I–V*) curves were collected at each point, each pilus filament was probed in at least three positions, and four to five pilus fibres were probed for each strain. Furthermore, the tip was periodically moved to the HOPG surface adjacent to the pili to control tip quality. From the linear portion of each *I–V* curve, we estimated the electrical resistance (*R*), as described elsewhere^28^, and used the average and s.d. of all the resistance values to compare the conductivity of the WT and Tyr3 pili.

### Data availability

The authors declare that the data supporting the findings of this study are available within the article and its Supplementary Information files, or from the corresponding author upon request.

## Additional information

**How to cite this article:** Steidl, R. J. *et al*. Mechanistic stratification in electroactive biofilms of *Geobacter sulfurreducens* mediated by pilus nanowires. *Nat. Commun.* 7:12217 doi: 10.1038/ncomms12217 (2016).

## Supplementary Material

Supplementary Information Supplementary Figures 1-6, Supplementary Tables 1-2 and Supplementary References
