# Corrigendum: Mechanistic stratification in electroactive biofilms of *Geobacter sulfurreducens* mediated by pilus nanowires

**DOI:** 10.1038/ncomms15474

**Published:** 2017-04-28

**Authors:** Rebecca J. Steidl, Sanela Lampa-Pastirk, Gemma Reguera

Nature Communications 7: Artilce number: 12217; DOI: 10.1038/ncomms12217 (2016); Published 08 02 2016; Updated 04 28 2017

Two previous studies (Vargas *et al*. 2013, Liu *et al*. 2014) reporting that conductive pili are required for long-range electron transport in *Geobacter sulfurreducens* were inadvertently omitted from the reference list of this Article, and should have been cited in the Introduction section where the possibility that pili function as biofilm electron carriers is discussed.

Vargas, M. *et al*. Aromatic amino acids required for pili conductivity and long-range extracellular electron transport in *Geobacter sulfurreducens*. *mBio* **4,** e00105-e00113 (2013).

Liu, X. *et al*. A *Geobacter sulfurreducens* strain expressing *Pseudomonas aeruginosa* type IV pili localizes OmcS on pili but is deficient in Fe(III) oxide reduction and current production. *Appl. Environ. Microbiol.* **80,** 1219-1224 (2014).

