# Supplementary material for: Mechanistic stratification in electroactive biofilms of Geobacter sulfurreducens mediated by pilus nanowires
Source: Nat Commun. 2016 Aug 2;7:12217. doi: 10.1038/ncomms12217 (PMC4974642; doi:10.1038/ncomms12217)
Supplement: Supplementary Information — Supplementary Figures 1-6, Supplementary Tables 1-2 and Supplementary References [file ncomms12217-s1.pdf]

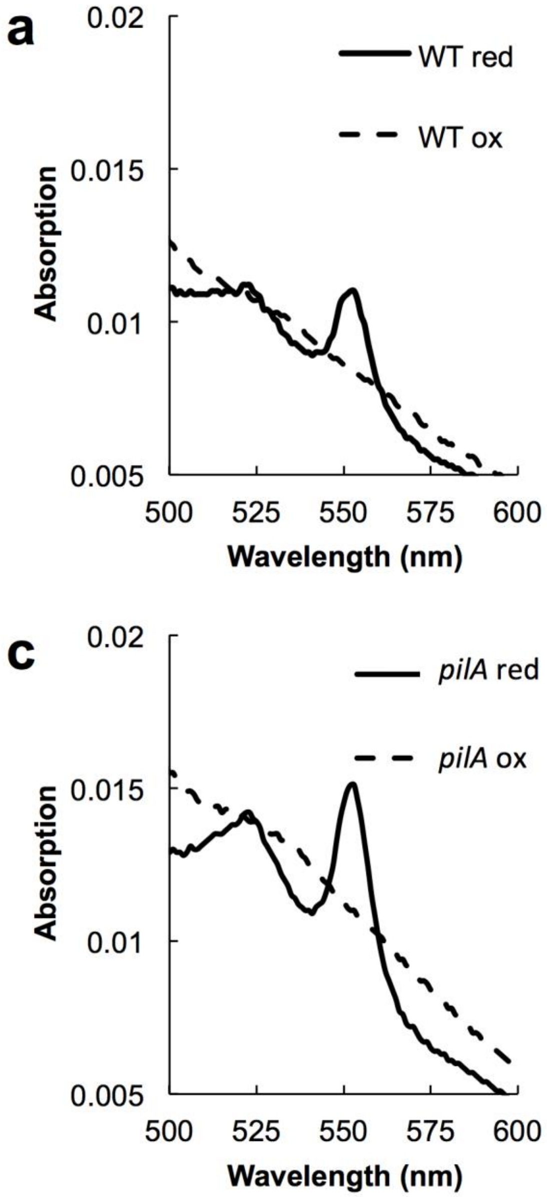


**Supplementary Fig. 1:** Representative absorption spectra of WT (**a**), *pilB* (**b**), *pilA* (**c**), and *pilA-E5A* (**d**) oxidized (dashed) and dithionite-reduced (solid) biofilm cell extracts, which were used to estimate their cytochrome content.

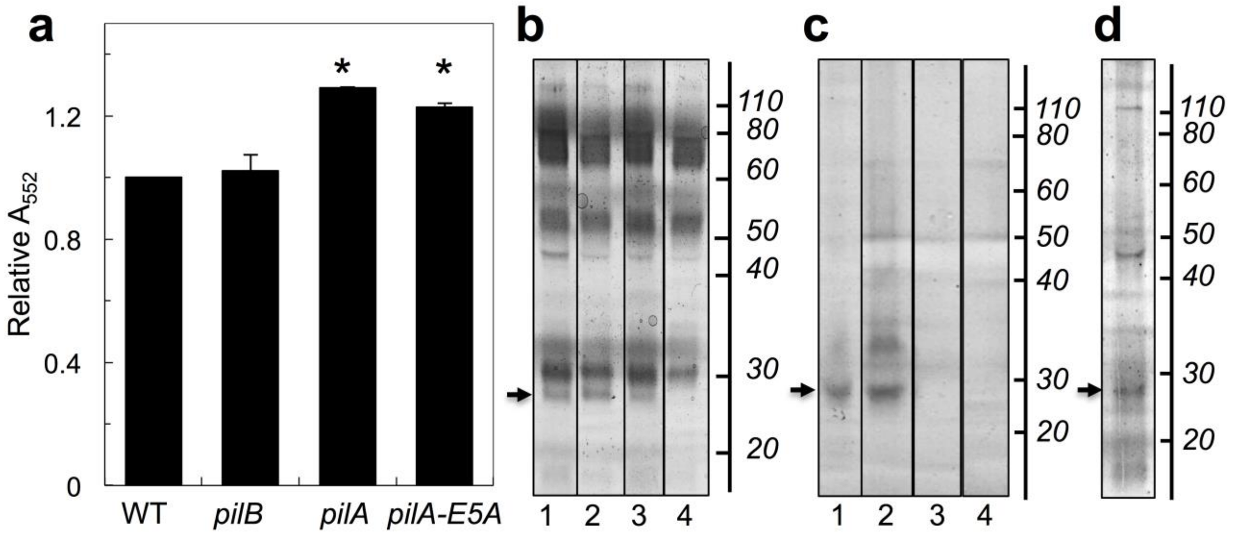


**Supplementary Fig. 2: Cytochrome expression in planktonic cells (a-b) and biofilms (c-d) of the WT and pili-deficient mutants. (a)** Cytochrome content (total cellular heme, measured with the pyridine hemochrome method) of planktonic cells of the pili-deficient strains *pilB*, *pilA*, and *pilA-E5A* relative to the WT. Shown are average and standard error of duplicate samples for each strain. Significant changes relative to the WT values were identified in *t*-test pairwise comparisons and are indicated with a star (* $p < 0.05$) (**b**) Heme-stained proteins in whole-cell extracts of planktonic cells of WT (lane 1), *pilB* (lane 2), *pilA*, (lane 3), and *omcZ* (lane 4), which was used as negative control lane for the ~ 30-kDa $OmcZ_S$ band (arrow). (**c-d**) Heme-stained proteins in the biofilm matrix of the WT, *pilB*, *pilA*, and *pilA-E5A* (lanes 1 to 4, respectively, in panel **c**), and Tyr3 (panel **d**). The arrows point at the migration of the $OmcZ_S$ band.

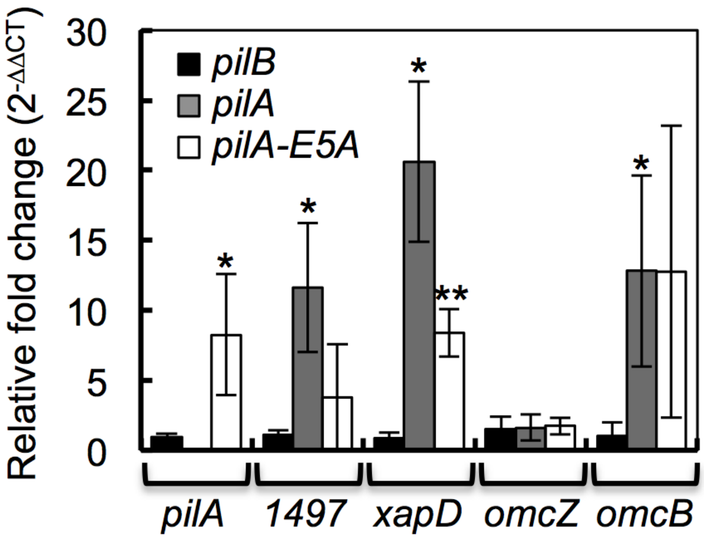


**Supplementary Fig. 3:** Expression of genes in the pilin operon (*pilA* and GSU1497), EPS synthesis (*xapD*), and outer membrane *c*-type cytochromes (*omcZ* and *omcB*) in 48-h old biofilms formed by the pili-deficient strains *pilB* (black), *pilA* (gray) and *pilA-E5A* (white) compared to WT. The constitutive gene *rpoD* was used as internal control. Data shown are average and standard deviation of four replicates. Statistically significant changes relative to the *pilB* values were identified in *t*-test pairwise comparisons and are indicated with one or two stars (* $p$ <0.05; ** $p$ <0.005)

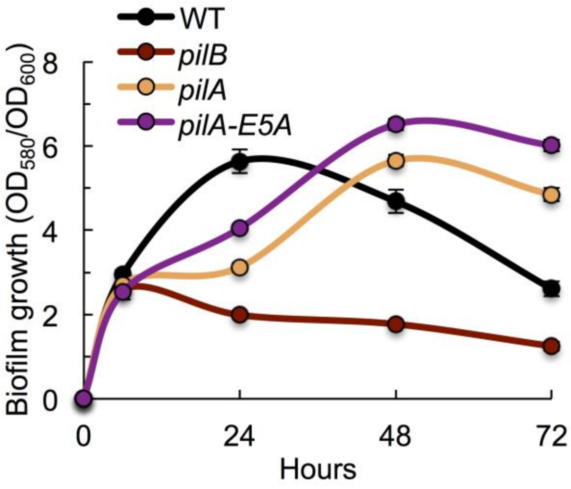


**Supplementary Fig. 4:** Time course biofilm formation assay showing biofilm growth ($OD_{580}$ of solubilized crystal violet associated with the biofilm normalized by $OD_{600}$ of the culture) of the WT, *pilB*, *pilA*, and *pilA-E5A*. Each time point shows the average and standard deviation of data collected for six replicate biofilm samples per strain.

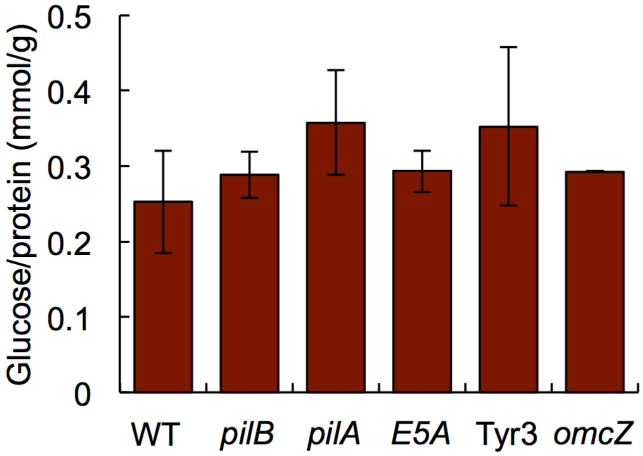


**Supplementary Fig. 5:** EPS content (as glucose equivalents, in mmol, per mg of cell protein) of biofilms of the WT, *pilB*, *pilA*, *pilA-E5A* (*E5A*), Tyr3, and *omcZ* strains. Shown are average and standard error of two independent experiments, each containing the matrix extracted from 48-h biofilm biomass collected from the wells of two 48-well plates.

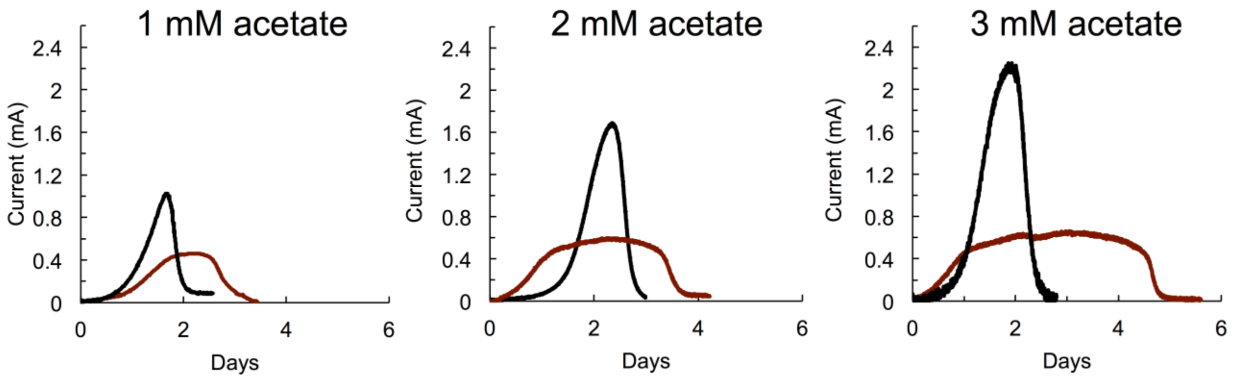


**Supplementary Fig. 6.** Representative plots of current generation by WT (black) and *pilB* (maroon) in MECs operated in batch and fed an initial concentration of 1, 2, or 3 mM acetate.

**Supplementary Table 1.** Strains and plasmids used in this study.

| Bacterial strain or plasmid | Relevant genotype and properties[a] | Source or reference(s) |
|---|---|---|
| ***Geobacter sulfurreducens*** | | |
| WT | Wild type strain PCA | [1] |
| *pilA* | Δ*pilA*::*aaaC1*, $Gm^r$ | This study |
| *pilB* | Δ*pilB*::*aaaC1*, $Gm^r$ | This study |
| *omcZ* | Δ*omcZ*::*aaaC1*, $Gm^r$ | This study |
| *pilA-E5A* | $pilA^{E5A}$::*aadA*, $Spec^r$ | This study |
| Δ*pilB* | Δ*pilB*::*loxP* | This study |
| *pilB*+ | Δ*pilB* complemented with pRG5-*pilB* | This study |
| *pilB gspE* | Δ*pilB* strain carrying the Δ*gspE*::*aaaC1* mutation, $Gm^r$ | This study |
| *pilB mshE* | Δ*pilB* strain carrying the Δ*mshE*::*aaaC1* mutation, $Gm^r$ | This study |
| Tyr3 | *pilA*Y27,32,57A::*aadA*, $Spec^r$ | This study |
| **Plasmids** | | |
| pCM351 | $Amp^r$, $Tet^r$, $Gm^r$, ColE1 *ori*, *oriT* | [2] |
| pCM158 | $Km^r$, *trfA*, *oriT*, *oriV*, ColE1 *ori*, *cre* | [2] |
| pCR2.1-TOPO | $Amp^r$, $Km^r$, ColE1 *ori* | Invitrogen |
| pRG5 | Shuttle vector for *G. sulfurreducens* $Spc^r$, $P_{taclac}$ | [3] |
| pRG5-*pilB* | pRG5 with *G. sulfurreducens pilB* | This study |

**Supplementary Table 2.** Primers used in this study.

| Primer | Sequence (5'-3') | Amplification | Use |
|---|---|---|---|
| RS1 | GATCTGGTCGGATACAACACC | *pilB* upstream | *pilB* |
| RS2 | TTATGCGGCCGCCATATGCATCTGCTA GCCTGCATAGACTCTCC | *pilB* upstream | *pilB* |
| RS3 | GTGTTAACCGGTCATATGCAGTGGCTG ACGACTAAACAAAATGCC | *pilB* downstream | *pilB* |
| RS4 | CTCTTGTGAGGATGCAGGTAC | *pilB* downstream | *pilB* |
| RS5 | ATGGACCTCGAAGCCTACCT | *pilA* upstream | *pilA* |
| RS6 | TTATGCGGCCGCCATATGCATCTGAAG CATAAGTG | *pilA* upstream | *pilA* |
| RS7 | GTGTTAACCGGTCATATGCACGCCCGA AAGTTAA | *pilA* downstream | *pilA* |
| RS8 | TCCATGCATCATTTTCGATG | *pilA* downstream | *pilA* |
| RS9 | ATATGGCGTTTACCGCAGAG | *omcZ* upstream | *omcZ* |
| RS10 | TTATGCGGCCGCCATATGCAGCTCCGA AGAAAGTCAAACG | *omcZ* upstream | *omcZ* |
| RS11 | GTGTTAACCGGTCATATGCAGATGCGC CAATCAGTACCTT | *omcZ* downstream | *omcZ* |
| RS12 | CACAGCCAGGTACCATCTGA | *omcZ* downstream | *omcZ* |
| RS13 | TTTCTCAGCAATCCATCGAG | *gspE* upstream | *pilB gspE* |
| RS14 | TTATGCGGCCGCCATATGCAATCTGTT CCATGTCGTGTGC | *gspE* upstream | *pilB gspE* |
| RS15 | GTGTTAACCGGTCATATGCAGTATGCC GACCTTCCGGTAT | *gspE* downstream | *pilB gspE* |
| RS16 | AGTGATTTTGCTCCGAATGG | *gspE* downstream | *pilB gspE* |
| RS17 | TTTCGGCCATGTACTCCTTT | *mshE* upstream | *pilB mshE* |
| RS18 | TTATGCGGCCGCCATATGCATCCTTGA CGATGCTTTCCAT | *mshE* upstream | *pilB mshE* |
| RS19 | GTGTTAACCGGTCATATGCACTCCTCC CTGCACGGTTGA | *mshE* downstream | *pilB mshE* |
| RS20 | CGACATCTTGTTCTCGTGGA | *mshE* downstream | *pilB mshE* |
| RS21 | TGCATATGGCGGCCGCATAA | *aaaC1 loxP* | Excible Gm marker |
| RS22 | TGCATATGACCGGTTAACAC | *aaaC1 loxP* | Excible Gm marker |
| RS23 | GTGGTGAAGGGGTAGGTTGA | *pilA* upstream | *pilA::aadA* |
| RS24 | GTTAGGCGTCATCCTGTGCTTAACTTT CGGGCGGATAGG | *pilA* upstream | *pilA::aadA* |
| RS25 | CAAGCCGACGCCGCTTCTTGATTAAAT ACATACTGGAGG | *pilA* downstream | *pilA*-Sp |
| RS26 | GCGACTTCCACTCGGTACC | *pilA* downstream | *pilA*-Sp |
| RS27 | GCACAGGATGACGCCTAAC | *aadA* | Sp marker |
| RS28 | GAAGCGGCGTCGGCTTG | *aadA* | Sp marker |
| RS29 | GGTTTCACCCTTATC**G<u>C</u>G**CTGCTGATC GTCGTT | *pilAE5A::aadA* | *pilA-E5A* |
| RS30 | AACGACGATCAGCAG**C<u>G</u>C**GATAAGGG TGAAACC | *pilAE5A::aadA* | *pilA-E5A* |

| | | | |
|---|---|---|---|
| RS31 | TCCGCAGTTCTCGGCG**<u>GC</u>T**CGTGTCAA GGCGTAC | *pilAY27A::aadA* | Tyr3 |
| RS32 | GTACGCCTTGACACG**A<u>GC</u>**CGCCGAGA ACTGCGGA | *pilAY27A::aadA* | Tyr3 |
| RS33 | GTATCGTGTCAAGGCG**<u>GC</u>C**AACAGCG CGGCGTC | *pilAY32A::aadA* | Tyr3 |
| RS34 | GACGCCGCGCTGTT**G<u>GC</u>**CGCCTTGAC ACGATAC | *pilAY32A::aadA* | Tyr3 |
| RS35 | CCGCATTTGCTGATGATCAAACC**<u>GC</u>T**C CGCCCGAAAGTTAA | *pilAY57A::aadA* | Tyr3 |
| RS36 | TTAACTTTCGGGCGG**A<u>GC</u>**GGTTTGATC ATCAGCAAATGCGG | *pilAY57A::aadA* | Tyr3 |
| RS37 | CCAACACAAGCAGCAAAAAG | *pilA* | qPCR |
| RS38 | GCAGCGAGAATACCGATGAT | *pilA* | qPCR |
| RS39 | ATGGGTGGCAAGGACTTTAC | GSU1497 | qPCR |
| RS40 | ACACCCGGTTACCAGAAGAG | GSU1497 | qPCR |
| RS41 | GTCCAACAAAGGGAACTGCT | *xapD* | qPCR |
| RS42 | CCTCCGCAGAGAGGTAATCA | *xapD* | qPCR |
| RS43 | CACGAGCCTGACACTCACTC | *omcZ* | qPCR |
| RS44 | AAGGTTGCTGACCTTGTTGG | *omcZ* | qPCR |
| RS45 | GACACGGTCAACCAGAACAA | *omcB* | qPCR |
| RS46 | GGTCCCAGTTTACGACAGGA | *omcB* | qPCR |
| RS47 | AGTTCTCGACGTACGCCACT | *rpoD* | qPCR |
| RS48 | TCAGCTTGTTGATGGTCTCG | *rpoD* | qPCR |

## Supplementary References


1 Caccavo, F., Jr. *et al. Geobacter sulfurreducens* sp. nov., a hydrogen- and acetate-oxidizing dissimilatory metal-reducing microorganism. *Appl. Environ. Microbiol.* **60**, 3752-3759 (1994).

2 Marx, C. J. & Lidstrom, M. E. Broad-host-range *cre-lox* system for antibiotic marker recycling in gram-negative bacteria. *Biotechniques* **33**, 1062-1067 (2002).

3 Kim, B. C. *et al.* OmcF, a putative *c*-type monoheme outer membrane cytochrome required for the expression of other outer membrane cytochromes in *Geobacter sulfurreducens*. *J. Bacteriol.* **187**, 4505-4513 (2005).
